# Uncertainty Relation for Errors Focusing on General POVM Measurements with an Example of Two-State Quantum Systems

**DOI:** 10.3390/e22111222

**Published:** 2020-10-27

**Authors:** Jaeha Lee, Izumi Tsutsui

**Affiliations:** 1 Institute of Industrial Science, The University of Tokyo, Chiba 277-8574, Japan; 2 Theory Center, Institute of Particle and Nuclear Studies, High Energy Accelerator Research Organization (KEK), Ibaraki 305-0801, Japan; izumi.tsutsui@kek.jp

**Keywords:** quantum foundations, quantum measurement, uncertainty relation

## Abstract

A novel uncertainty relation for errors of general quantum measurement is presented. The new relation, which is presented in geometric terms for maps representing measurement, is completely operational and can be related directly to tangible measurement outcomes. The relation violates the naïve bound ℏ/2 for the position-momentum measurement, whilst nevertheless respecting Heisenberg’s philosophy of the uncertainty principle. The standard Kennard–Robertson uncertainty relation for state preparations expressed by standard deviations arises as a corollary to its special non-informative case. For the measurement on two-state quantum systems, the relation is found to offer virtually the tightest bound possible; the equality of the relation holds for the measurement performed over every pure state. The Ozawa relation for errors of quantum measurements will also be examined in this regard. In this paper, the Kolmogorovian measure-theoretic formalism of probability—which allows for the representation of quantum measurements by positive-operator valued measures (POVMs)—is given special attention, in regard to which some of the measure-theory specific facts are remarked along the exposition as appropriate.

## 1. Introduction

Since its advocation nearly a century ago, the uncertainty principle has unquestionably stood as one of the foundational pillars of quantum mechanics, marking the indeterministic nature of the microscopic world. Soon after Heisenberg’s seminal exposition [1] in 1927, the first mathematical formulation of the principle was presented by Kennard [2], revealing the lower bound ℏ/2 of the product of the standard deviations of the canonically conjugate pair of the position and momentum. Inspired by Weyl’s alternative and more modern proof [3] of Kennard’s finding utilizing the Cauchy–Schwarz inequality, Robertson subsequently obtained its generalization [4] to the product of the standard deviations

(1) σ(A)σ(B)≥|〈[A,B]〉|/2

of arbitrary observables *A* and *B*, with the lower bound being characterized by the absolute value of the expectation value of the commutator [A,B]:=AB−BA. Owing to its mathematical clarity and broad applicability, the Kennard–Robertson inequality became a standard textbook material as a succinct expression of quantum indeterminacy.

Nevertheless, the Kennard–Robertson relation embraced very little, if any, notion of quantum measurement in the strict sense, given the fact that Heisenberg—even though his own conception of uncertainty (or ‘indeterminateness’ [5]) is difficult to precisely pin down from the rather nebulous depiction in his writings—did entertain concepts of error and disturbance pertaining to quantum measurement, which he exemplified with several physical models including the famous gamma-ray microscope Gedankenexperiment. This rather unsatisfactory status encouraged the development of alternative formulations of uncertainty relations incorporating measurement.

A popular model for the description of quantum measurement has been the indirect measurement scheme, which explicitly considers an external quantum system of an ancillary meter device in addition to the original quantum system of interest, thereby allowing for a physically intuitive representation of an otherwise obscure measurement process. The Arthurs–Kelly–Goodman relations [6,7] and the more recent Ozawa relations [8,9], along with their refinement [10] and modifications [11,12], are among the most notable formulations that are founded on this model, whereby their formulation of error (and disturbance) defined for measurements associated with positive-operator valued measures (POVMs) over the real field admits an intelligible representation. Apart form these, uncertainty relations have also been framed on the foundation of estimation theory [13,14] in addition to having been formulated from a measure-theoretic viewpoint [15,16,17].

In this paper, a novel uncertainty relation that marks the trade-off relation between the errors of quantum measurement is presented. The relation, being established upon the conceivably simplest and most general framework of measurement, is valid for all quantum measurements of statistical nature. Notably, it is formulated without reference to any specific measurement models whatsoever, whereby the only objects required are the tangible measurement outcomes; this specifically entails that the relation is operationally verifiable, the fact of which is in contrast to some alternative formulations (including Ozawa’s) that generally require objects that the outcomes alone cannot dictate, as having been pointed out by several authors [15,18].

Interestingly, the new relation is found to generically violate the naïve lower bound |〈[A,B]〉|/2 prescribed by the commutator of the observables to be measured, which is in line with the recent similar findings espoused notably by Ozawa [9]. Nevertheless, Heisenberg’s spirit of the uncertainty principle still stands strong, albeit perhaps in a laxer and more qualitative form than is commonly conceived or was originally intended.

Beyond the orthodox relations regarding quantum indeterminacy, error, and disturbance, the uncertainty principle was also found to be accountable for various forms of incompatibilities of diverse nature, such as time and energy [19,20,21,22,23], entropy [24,25,26,27,28], conservation law [29,30,31,32], speed limit [33,34,35,36,37,38,39,40], gate implementation [41,42], and counterfactuality [43,44,45,46]. In this regard, the new relation is found to entail the standard Kennard–Robertson relation as a corollary to it, thereby providing a seamless connection between the two different realms of quantum uncertainty. The physical ramifications and a comprehensive mathematical description of the new universal formulation [47] shall be presented in later publication; there, other notable formulations, including the Arthurs–Kelly–Goodman and the Ozawa relations, are also accounted for from its perspective.

This paper is intended as an extended paper of the previous concise report [48], to which the reader is referred as appropriate. In this regard, this paper supplements the exposition by providing additional remarks on the POVM formalism of quantum measurements, which is the most common and standard method to describe general quantum measurements based on the Kolmogorovian framework of probability. An analysis of the measurement on two-state quantum systems is also included, which serves to exemplify the general results with a simple and concrete model, and also illustrates the technical advantage of the new relation over Ozawa’s in terms of tightness.

This paper is organized as follows. Section 2 offers a succinct exposition of the essential tools; the basic notions regarding measurement, such as quantum- and classical-state spaces, quantum measurements, and their dual notions are first introduced, which are then found to point to the existence of an adjoint pair of state-dependent maps, termed the pullback and the pushforward. In Section 3, the error of quantum measurement is introduced, followed by a useful equivalence condition on which a measurement becomes free from it. The main result, i.e., the new uncertainty relation for errors, is then presented in Section 4; the relation is subsequently found to have an important connotation regarding the errors of two incompatible (non-commutative) quantum observables, whereby Heisenberg’s philosophy of the uncertainty principle is revisited. In Section 5, the new relation is then found to entail the Kennard–Robertson relation as a simple corollary, thereby attaining a seamless unification of the two different realms of quantum uncertainty. In Section 6, the relation is examined through a simple example regarding measurements on two-state quantum systems. Here, the analytical formulae for the errors and the lower bound of the relation are given, whereby the validity of the relation is confirmed and the necessary and sufficient conditions for the equality to hold is characterized. The final Section 7 is devoted to discussions in perspective of the previous studies, where the Ozawa relation for errors of quantum measurement is examined.

## 2. Pullback and Pushforward of a Quantum Measurement

A quantum measurement is found to induce an adjoint pair of local (i.e., state-dependent) maps between the quantum- and classical-observable spaces. The pair, termed the pullback and the pushforward of a quantum measurement, plays an essential role.

### 2.1. Quantum Measurement and Its Adjoint

**Figure 1:**
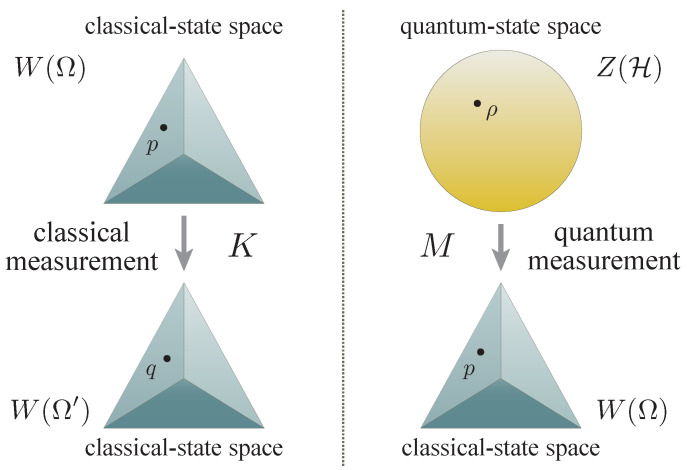
The basic premise of classical and quantum measurements. The space of quantum states Z(H) is depicted as a ball, whereas the space of probability distributions W(Ω) is represented by a tetrahedron. They each offer symbolic graphical representations of the respective state spaces; the former 3-dimensional unit ball can be identified with the state space of a 2-dimensional quantum system as a collection of all the Bloch vectors, whereas the latter 3-dimensional probability simplex can be identified with the set of all probability distributions on the sample space consisting of 4 elements. (**Left**) A classical measurement *K* can be considered as an affine map $K:W\left(\Omega \right)\to W\left(\Omega^{\prime }\right)$, for indeed in any classical measurement of statistical nature, the end result of the measurement should be a probability distribution $q=Kp\in W(\Omega^{\prime })$ on the set $\Omega^{\prime }$ of possible outcomes, the profile of which may depend on the state p∈W(Ω) of the system over which the measurement is performed. (**Right**) In the same vein, an affine map M:Z(H)→W(Ω) is interpreted as a quantum measurement, which yields a probability distribution p=Mρ∈W(Ω) on the set Ω of possible outcomes, the profile of which is dependent on the choice of the quantum state ρ∈Z(H) over which the measurement is performed.

Let Z(H) denote the state space of a quantum system, which is hereafter modeled as the convex set of all the density operators ρ on a Hilbert space H. Its classical counterpart W(Ω) is modeled as the convex set of all the probability distributions *p* on a sample space Ω. The primary objects of interest are the affine maps *M* from quantum-state spaces to classical-state spaces, which are understood as quantum measurements in this paper (see Figure 1). One should indeed find this interpretation reasonable, for the archetypal projection measurement induces such a map. In general, if one adopts Kolmogorov’s measure-theoretic formalism [49] to model probability, the map *M* admits a familiar representation in terms of POVMs [50]. In this paper, special attention is given to this most common and standard formalism of modeling probability, thereby making comments on subjects and facts that are measure-theory specific along the discourse as appropriate.

An important observation is that a quantum measurement *M* induces a natural map $M^{\prime }$, termed its adjoint, which takes a classical function on Ω to a Hilbert-space operator on H. This dual notion of a quantum measurement is uniquely characterized by the relation M′fρ*=fMρ*, which holds for all complex functions *f* on Ω and quantum states ρ on H. Here, the shorthand ${\langle X\rangle }_{\rho }:=\mathrm{Tr}\left[X\rho \right]$ is defined for a pair of a Hilbert-space operator *X* and a density operator ρ on H, whereas ${\langle f\rangle }_{p}:=\int_{\Omega }f\left(\omega \right)p\left(\omega \right)\;d\omega$ is defined for a pair of a complex function *f* and a probability distribution *p* on Ω.

### 2.2. The Space of Observables

The space of quantum observables is hereafter modeled by the linear space S(H) of all the self-adjoint operators on a Hilbert space H. Here, each quantum state ρ∈Z(H) defines a seminorm ${∥A∥}_{\rho }:=\;\sqrt{{\langle A^{†}A\rangle }_{\rho }}$, A∈S(H) on the space, thereby inducing a natural equivalence relation $A{∼}_{\rho }B⟺{∥A-B∥}_{\rho }=0$ on it. This allows for the classification of all the quantum observables into their equivalence classes ${\left[A\right]}_{\rho }:=\left\{B\in S\left(H\right):A{∼}_{\rho }B\right\}$, which collectively constitute the quotient space $S\left(H\right)/{∼}_{\rho }$, the completion of which is hereafter denoted by $S_{\rho }\left(H\right)$. In the same vein, a probability distribution p∈W(Ω) induces a seminorm ${∥f∥}_{p}:=\sqrt{{\langle f^{†}f\rangle }_{p}}$ on the linear space R(Ω) of all the real functions *f* defined on the sample space Ω. The identification $f{∼}_{p}g⟺{∥f-g∥}_{p}=0$ results in the classification of the real functions into their equivalence classes ${\left[f\right]}_{p}:=\left\{g\in R\left(\Omega \right):f{∼}_{p}g\right\}$, which collectively make up the quotient space $R\left(\Omega \right)/{∼}_{p}$, further leading to its completion $R_{p}\left(\Omega \right)$. Here, the adjoint $A^{†}$ of a Hilbert-space operator and the complex conjugate $f^{†}$ of a complex function are introduced to expose the structure of the seminorms so that they respectively admit obvious extensions beyond self-adjoint operators and real functions. As commonly practiced, with a slight abuse of notation, the equivalence classes are hereafter denoted by one of their representatives.

Note that the norm on $S_{\rho }\left(H\right)$ admits a unique inner product ${\langle A,B\rangle }_{\rho }:={\langle \left\{A,B\right\}\rangle }_{\rho }/2$ characterized by the anti-commutator {A,B}:=AB+BA that reproduces the original norm ${∥A∥}_{\rho }^{2}={\langle A,A\rangle }_{\rho }$. The same remark also goes for the inner product ${\langle f,g\rangle }_{p}:={\langle fg\rangle }_{p}$ defined on $R_{p}\left(\Omega \right)$ that satisfies ${∥f∥}_{p}^{2}={\langle f,f\rangle }_{p}$.

### 2.3. Pullback and Pushforward

Given a quantum measurement M:Z(H)→W(Ω), a crucial observation regarding its adjoint is the validity of the inequality

(2) $${∥f∥}_{M\rho }\geq {∥M^{\prime }f∥}_{\rho }$$

for any quantum state ρ on H and complex function *f* on Ω. In view of its equivalence to the condition $\sigma_{M\rho }\left(f\right)\geq \sigma_{\rho }\left(M^{\prime }f\right)$, where the symbols $\sigma_{\rho }\left(A\right):=\sqrt{{∥A∥}_{\rho }^{2}-{\langle A\rangle }_{\rho }^{2}}$ and $\sigma_{p}\left(f\right):=\sqrt{{∥f∥}_{p}^{2}-{\langle f\rangle }_{p}^{2}}$ respectively denote the quantum and classical standard deviations, the inequality (2) admits a physically intuitive and operational interpretation as a statement regarding the lower bound of the efficiency of quantum measurements in estimating the expectation value of an observable $A=M^{\prime }f$ by means of an estimator function *f* through the measurement *M*. As is expounded in [47], the inequality (2) can be understood as a corollary to the Kadison–Schwarz inequality [51]; if one adopts the measure-theoretic formalism to model probability, thereby allowing for the representation of the quantum measurement *M* by POVM, Naimark’s dilation theorem [52,53] becomes directly applicable to provide a tailored and more concrete proof.

A direct connotation of the inequality (2) is the implication $f{∼}_{M\rho }g⟹M^{\prime }f{∼}_{\rho }M^{\prime }g$ for each quantum state ρ∈Z(H). This allows for the adjoint $M^{\prime }$ to be understood as the map between the quotient spaces, thereby pointing to the existence of the map

(3) $$M_{\;\rho }^{*}:R_{M\rho }\left(\Omega \right)\to S_{\rho }\left(H\right)$$

and its adjoint

**Figure 2:**
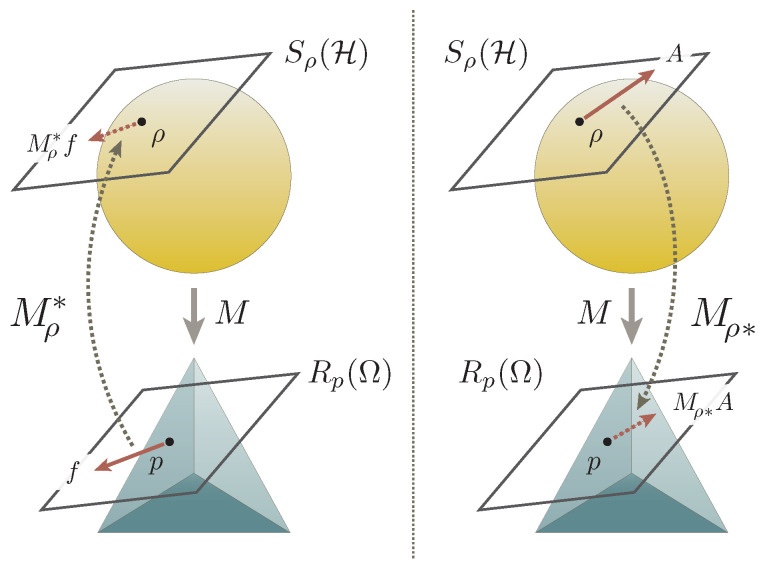
The pullback and the pushforward of a quantum measurement. (**Left**) A quantum measurement *M* induces the pullback $M_{\;\rho }^{*}$ from the linear spaces $R_{p}\left(\Omega \right)$ to $S_{\rho }\left(H\right)$, each of which is attached to the respective points p=Mρ∈W(Ω) and ρ∈Z(H) of the corresponding state spaces. (**Right**) The measurement *M* also entails the pushforward that maps the observables in the opposite direction. The pullback and the pushforward are dual notions to one another, being connected by the relation (5). They are both non-expansive maps—the norm either decreases or remains unchanged under their actions—that also preserve the expectation values.

(4) Mρ**:Sρ(H)→RMρ(Ω),

which are hereafter called the pullback and the pushforward of the measurement *M* over the quantum state ρ, respectively (see Figure 2); the pullback and the pushforward are dual notions to one another, characterized by the relation

(5) A,Mρ*fρ=Mρ**A,fMρ

that is valid for all $A\in S_{\rho }\left(H\right)$ and $f\in R_{M\rho }\left(\Omega \right)$. By construction, one finds that both the pullback ${∥f∥}_{M\rho }\geq {∥M_{\;\rho }^{*}f∥}_{\rho }$ and the pushforward ${∥A∥}_{\rho }\geq {∥M_{\;\rho *}A∥}_{M\rho }$ are non-expansive, in addition to the fact that they preserve the expectation values ${\langle f\rangle }_{M\rho }={\langle M_{\;\rho }^{*}f\rangle }_{\rho }$ and ${\langle A\rangle }_{\rho }={\langle M_{\;\rho *}A\rangle }_{M\rho }$.

If one adopts the Kolmogorovian formalism of probability, the pushforward (4) admits a concrete expression in terms of the standard tools of measure and integration theory. While the topic is beyond the scope of this paper, a concise description of the result that nevertheless becomes relevant in later sections is included; as is presented in [47], the formula for the pushforward $M_{\;\rho *}A=d\nu_{\rho }/d\mu_{\rho }$ is furnished by the Radon–Nikodým derivative of the signed measure $\nu_{\rho }\left(\Delta \right):=\;\left({∥A+\;M_{\;\rho }^{*}\chi_{\Delta }∥}_{\rho }^{2}-\;{∥A-M_{\;\rho }^{*}\chi_{\Delta }∥}_{\rho }^{2}\right)/4$ with respect to the probability measure $\mu_{\rho }:=M\rho$, where $\chi_{\Delta }$ represents the measurable characteristic function (*alias* indicator function) of a measurable set Δ pertaining to the σ-algebra on the sample space Ω.

## 3. Error of a Quantum Measurement

Based on the tools introduced so far, the *error* regarding a quantum measurement *M* of an observable *A* over a state ρ is defined as the amount of contraction

(6) ερ(A;M):=∥A∥ρ2−∥Mρ**A∥Mρ2

induced by the pushforward regarding the observable of one’s concern. Non-negativity $\varepsilon_{\rho }\left(A;M\right)\geq 0$ of the error follows immediately from the non-expansiveness of the pushforward, whereas the homogeneity $\varepsilon_{\rho }\left(tA;M\right)=\left|t\right|\;\varepsilon_{\rho }\left(A;M\right)$, ∀t∈R and the subadditivity $\varepsilon_{\rho }\left(A;M\right)+\varepsilon_{\rho }\left(B;M\right)\geq \varepsilon_{\rho }\left(A+B;M\right)$ can also be readily confirmed. In other words, the error (6) furnishes a seminorm on the ‘tangent’ space $S_{\rho }\left(H\right)$ of quantum observables attached to the state ρ over which the measurement is performed.

The error (6) admits an operational interpretation as the minimal error of the local reconstruction of a quantum observable through the measurement. In this regard, let $\varepsilon_{\rho }\left(A;M,f\right):=\sqrt{{∥A-M_{\;\rho }^{*}f∥}_{\rho }^{2}+\left({∥f∥}_{M\rho }^{2}-{∥M_{\;\rho }^{*}f∥}_{\rho }^{2}\right)}$ provide an evaluation of the precision of the reconstruction of an observable *A* by means of the pullback $M_{\;\rho }^{*}f$, which is in turn created from an estimator *f* through the measurement *M*; in this paper, this is called the error with respect to the estimator *f* (*abbr*. *f*-error). A simple computation using (5) reveals the decomposition of its square ερ(A;M,f)2=ερ(A;M)2+∥Mρ**A−f∥Mρ2 into the sum of the squares of the error (6) and the estimation error, thereby pointing to an operational characterization of the error (6) as the minimum of the *f*-error over all the local estimators, as well as the interpretation of the pushforward as the unique locally optimal estimator that realizes it.

It is useful to identify the conditions on which the measurement becomes free from error. In what follows, a quantum measurement *M* is said to be capable of an *errorless measurement* of *A* over ρ, if the error (6) vanishes. In this regard, one finds the equivalence of the following two conditions:

(7) ερ(A;M)=0⟺A=Mρ*Mρ**A.

The implication ⟹ follows from ερ(A;M)≥∥A−Mρ*Mρ**A∥Mρ, whereas the converse ⟸ is due to ∥A∥ρ≥∥Mρ**A∥Mρ≥∥Mρ*Mρ**A∥ρ, the evaluations of which are both direct consequences of the non-expansiveness of the pullback and the pushforward.

An important fact is that an errorless measurement of an observable A∈S(H) is always available; the projection measurement *M* associated with it is capable of such a measurement (not just locally over a certain quantum state, but in fact globally over every state). To see this, it suffices to realize that the pushforward of an observable *A* by the projection measurement *M* associated with it becomes the identity function $\left(M_{\;\rho *}A\right)\left(a\right)=a$ on the spectrum of *A*; one may directly derive the result from the formula for the pushforward described above, but can also—perhaps more readily—confirm this from the fact that the identity function indeed fulfills the characterization (5) of the pushforward.

## 4. Uncertainty Relation for Errors and the Uncertainty Principle

Now that the necessary tools have been introduced, the main result, i.e., the new uncertainty relation for errors, is presented. Its implication regarding the uncertainty principle is also investigated.

### 4.1. The Uncertainty Relation for Errors

Let *A* and *B* be an arbitrary pair of quantum observables of the system H, and let ρ∈Z(H) be a quantum state of one’s choice. Then, for any quantum measurement M:Z(H)→W(Ω), the inequality

(8) $$\varepsilon_{\rho }\left(A;M\right)\;\varepsilon_{\rho }\left(B;M\right)\geq \sqrt{R^{2}+I^{2}}$$

holds with

(9) R:={A,B}2ρ−Mρ**A,Mρ**BMρ

and

(10) I:=[A,B]2iρ−[Mρ*Mρ**A,B]2iρ−[A,Mρ*Mρ**B]2iρ.

This can be obtained through a simple application of the renowned Cauchy–Schwarz inequality over the semi-inner product $\langle \left(X,f\right),\left(Y,g\right)\rangle :={\langle X^{†}Y\rangle }_{\rho }+{\langle f^{†}g\rangle }_{M\rho }-{\langle M^{\prime }f^{†}M^{\prime }g\rangle }_{\rho }$ defined for the products of Hilbert-space operators and complex functions. Indeed, in view of the fact that $\varepsilon_{\rho }\left(A;M\right)=\sqrt{\langle \left(X_{A},f_{A}\right),\left(X_{A},f_{A}\right)\rangle }$ with the shorthands XA:=A−Mρ*Mρ**A and fA:=Mρ**A, the product of the errors of the two observables *A* and *B* are found to be bounded from below by the absolute value of the complex number $\langle \left(X_{A},f_{A}\right),\left(X_{B},f_{B}\right)\rangle$, whose real part R is given by (9) whereas the imaginary part I by (10).

From a mathematical (geometric) point of view, the real part R in (9) represents the decrease of the induced metric on the bundle of ‘localized’ quantum observables, whose loss being inevitably caused by the quantum measurement *M*. In fact, this term is also found to be shared with the inequality regarding the errors of classical measurements $K:W\left(\Omega \right)\to W\left(\Omega^{\prime }\right)$, which are defined as affine maps between classical-state spaces (see Figure 1). This fact reveals that the semiclassical contribution R of the lower bound is shared in common between both classical and quantum measurements, thereby suggesting that it is not necessarily of quantum origin; a more comprehensive account of this subject is found in [47]. On the other hand, the imaginary part I in (10), which consists of three commutators and imposes an additional constraint on the attainable lower bound of the product of the errors, marks the essence of quantum measurements. In view of this, the simplified form

(11) $$\varepsilon_{\rho }\left(A;M\right)\;\varepsilon_{\rho }\left(B;M\right)\geq \left|I\right|$$

of the relation (8) should be mostly adequate to account for the distinguishing characteristics of quantum theory.

### 4.2. The Uncertainty Principle

The uncertainty relation (8) implies that the product of the errors may potentially violate the naïve bound $|{\langle \left[A,B\right]\rangle }_{\rho }|/2$. In fact, the violation is always available for any pair of quantum observables *A* and *B*, for indeed, an errorless measurement of either observable—this is always available (see Section 3)—trivially violates it. Nevertheless, Heisenberg’s philosophy regarding the uncertainty principle still remains valid, albeit perhaps in a weaker form than is originally intended; the uncertainty relation (8) forbids a simultaneous errorless measurement of a pair of quantum observables *A* and *B* whenever the term ${\langle \left[A,B\right]\rangle }_{\rho }$ does not vanish. Indeed, if there were such a measurement *M*, the relation (8) combined with the equivalence (7) of the two conditions of the errorless measurement would lead to a contradiction $0\geq \sqrt{{\left|0\right|}^{2}+{\left|{\langle \left[A,B\right]\rangle }_{\rho }/2i\right|}^{2}}$. It is to be emphasized here that one of the errors may vanish as long as the other does not; the crux is that both errors cannot vanish together.

An immediate consequence of this no-go theorem is that, for non-trivial quantum systems (i.e., quantum systems whose Hilbert spaces have dimensions of no less than two), there exists no quantum measurement that is capable of singlehandedly measuring every observable errorlessly over every state.

## 5. Quantum Indeterminacy

An interesting observation is that the Kennard–Robertson relation for quantum indeterminacy actually emerges as a trivial case of the relation (8) for measurement errors as the notion of measurement fades towards the limit of non-informativeness; one thus attains a seamless unification of the two different realms of quantum uncertainty regarding measurement error and state indeterminacy.

One may call a quantum measurement *M trivial*, or *non-informative*, when it is a constant map between the state spaces, i.e., when there exists some fixed probability distribution $p_{0}\in W\left(\Omega \right)$ for which $p_{0}=M\rho$ holds for every ρ∈Z(H). The pullback and the pushforward of a non-informative measurement *M* are found to be respectively characterized by the identity operator $M_{\;\rho }^{*}f={\langle f\rangle }_{M\rho }$ on the Hilbert space H and the constant function Mρ**A=〈A〉ρ on the sample space Ω, each weighted by the expectation values of the elements concerned. Non-informativeness of the measurement thus reduces the error (6) to the standard deviation $\varepsilon_{\rho }\left(A;M\right)=\sigma_{\rho }\left(A\right)$, thereby bringing the relation (8) further towards

(12) $$\sigma_{\rho }\left(A\right)\;\sigma_{\rho }\left(B\right)\geq \frac{1}{2}\sqrt{{\left|{\langle \left\{A,B\right\}\rangle }_{\rho }-2\;{\langle A\rangle }_{\rho }{\langle B\rangle }_{\rho }\right|}^{2}+{\left|{\langle \left[A,B\right]\rangle }_{\rho }\right|}^{2}}.$$

In fact, this inequality is known as the Schrödinger relation [54], from which the Kennard–Robertson relation (1) follows immediately by disregarding the first term in the square-root that appears in the R.H.S.; note that this procedure directly corresponds to the reduction of the relation (8) to its simplified form (11) by omitting the semiclassical contribution R of the lower bound.

As is presented in [47], the reduction of the relation (8) to the Schrödinger relation (12) may also be found in non-trivial measurements as well, albeit locally in general. One such condition that becomes relevant to this paper is when the measurement outcomes are concentrated at a single element, i.e., when the measurement over a quantum state ρ∈Z(H) happens to result in the delta distribution $M\rho =\delta_{\omega }$ concentrated at some point ω∈Ω. An illustrative example of this would be the projection measurement performed over the eigenstates of the ‘measurement observable’ associated with it; more explicitly, given an eigenstate $\rho =\psi_{m}$ of an eigenvalue m∈R of the ‘measurement observable’ $\hat{M}$ (i.e., $\hat{M}\psi_{m}=m\psi_{m}$), the projection measurement *M* associated with it performed over $\psi_{m}$ results in the probability distribution $M\psi_{m}=\delta_{m}$ as desired.

## 6. Example: Measurement on Two-State Quantum Systems

As a simple demonstration of the uncertainty relation (8), the measurement on two-state quantum systems is investigated. Since a comprehensive study of the general case of such a measurement, which shall be given elsewhere, is beyond the scope of this paper, the analysis is confined to a specific setting that nevertheless marks its essence. For definiteness, the target observables to be measured are set to the *x* and *y* components of the familiar spin-1/2 angular momentum. The analysis consists of two parts; as the archetype of non-trivial measurements, the projection measurement associated with the remaining *z* component of the spin angular momentum is investigated, followed by the analysis of trivial measurements. Specifically, one finds that the relation offers virtually the tightest lower bound possible; in both trivial and non-trivial cases, the equality of the relation holds for the measurement over all pure states.

### 6.1. Preparation of the Symbols

As is often the case with the study of two-state quantum systems $H≃C^{2}$, the Pauli matrices

(13) $$\sigma_{x}:=\left(\begin{array}{cc} 0 & 1\\ 1 & 0 \end{array}\right),\;\sigma_{y}:=\left(\begin{array}{cc} 0 & -i\\ i & 0 \end{array}\right),\;\sigma_{z}:=\left(\begin{array}{cc} 1 & 0\\ 0 & -1 \end{array}\right)$$

are found to serve as a convenient tool for the analysis. The triplet $\sigma :=(\sigma_{x},\sigma_{y},\sigma_{z})$ together with the identity operator Id allows for the unique representation

(14) $$\rho =\frac{1}{2}\left(\mathrm{Id}+r\cdot \sigma \right)\in Z\left(H\right)$$

of quantum states ρ∈Z(H) by the Bloch vectors $r:=\left(r_{x},r_{y},r_{z}\right)\in R^{3}$, |r|≤1, where the standard convention $r\cdot \sigma :=\sum r_{i}\sigma_{i}$ is adopted. The spin-1/2 angular momentum S:=(ℏ/2)σ then admits a familiar representation

(15) $$S_{x}:=\frac{\hbar }{2}\;\sigma_{x},\;S_{y}:=\frac{\hbar }{2}\;\sigma_{y},\;S_{z}:=\frac{\hbar }{2}\;\sigma_{z}$$

along the respective Cartesian axes. For definiteness, the target observables to be measured are fixed to $A:=S_{x}$ and $B:=S_{y}$ throughout this section.

### 6.2. Non-Trivial Projection Measurement

Here, non-trivial measurements on two-state quantum systems are investigated. For the purpose of this paper, the analysis is confined (without much loss of generality) to projection measurements, which are the archetypes of all possible measurements that can be performed on a quantum system. Specifically, the projection measurement associated with the ‘measurement observable’ $S_{z}$ is chosen for this demonstration, in which the sample space $\Omega =\{m_{-},m_{+}\}$ of the measurement outcomes is the two-element set consisting of its two distinct eigenvalues $m_{\pm }=\pm \;\hbar /2$.

#### 6.2.1. Errors of the Measurement and the Uncertainty Principle

As a first step, the computation of the errors of the measurement of the target observables is conducted. In this regard, the pushforwards of the target observables $S_{x}$ and $S_{y}$ by the projection measurement *M* associated with $S_{z}$, respectively, read

(16) (Mρ**Sx)(m)={ℏ2·rx1+rzm=+ℏ2ℏ2·rx1−rzm=−ℏ2

and

(17) (Mρ**Sy)(m)={ℏ2·ry1+rzm=+ℏ2ℏ2·ry1−rzm=−ℏ2

over the state $\rho ≃r=(r_{x},r_{y},r_{z})$, where the Bloch-vector representation (14) of quantum states is adopted with the convention 0/0:=0 hereafter; as always, the above results (16) and (17) can be directly computed from the formula for the pushforward, but the fact that they are indeed the pushforwards of $S_{x}$ and $S_{y}$, respectively, can also be readily confirmed by their characterization as the unique functions that satisfy the relation (5). The errors of the measurement of the respective observables

(18) $$\varepsilon_{\rho }\left(S_{x};M\right)=\frac{\hbar }{2}\;\sqrt{1-\frac{r_{x}^{2}}{1-r_{z}^{2}}}$$

and

(19) $$\varepsilon_{\rho }\left(S_{y};M\right)=\frac{\hbar }{2}\;\sqrt{1-\frac{r_{y}^{2}}{1-r_{z}^{2}}}$$

can thus be obtained by simple substitution of the pushforwards appearing in the definition of the error (6) with the functions (16) and (17).

**Figure 3:**
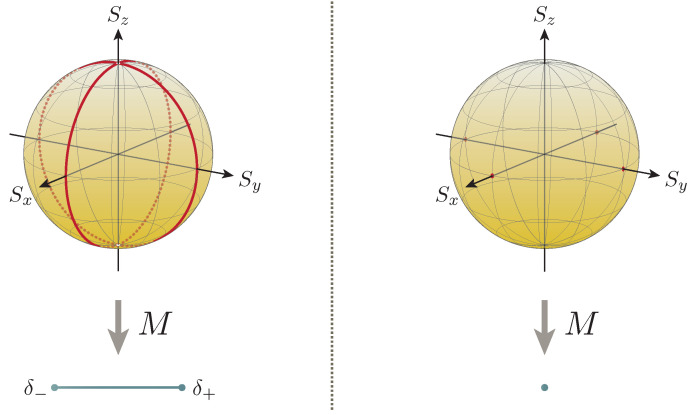
Measurement on two-state quantum systems. (**Left**) A dichotomic measurement on two-state quantum systems can be represented by an affine map from a 3-dimensional unit ball to a line segment; for the current example, the extreme points $\delta_{\pm }$ of the line segment are the delta measures concentrated at the respective eigenvalues ±ℏ/2 of the ‘measurement observable’ $S_{z}$. The collections of all the quantum states over which the measurements of $S_{x}$ and $S_{y}$ are free from error take the shape of the respective orthodomes (discounting the north and south poles) (20) and (21) colored in red. Note that these two sets have empty intersection, which indeed attests to the (weaker form of the) uncertainty principle forbidding the errors from vanishing simultaneously, unless the expectation value of the commutator vanishes (this happens if and only if the state belongs to the unique cross-section of the ball containing the equator). The equality of the relation holds over all the sphere. (**Right**) A trivial measurement can be reduced to a constant map to a singleton. The errors (i.e., standard deviations) of the observables $S_{x}$ and $S_{y}$ vanish if and only if the measurement is performed over their respective eigenstates depicted by the red dots. Again, the equality of the relation holds over all the sphere.

At this point, one finds that the errorless measurements of the respective observables are attainable precisely over the quantum states

(20) $$\varepsilon_{\rho }\left(S_{x};M\right)=0⟺\rho ≃r\in \left\{\left(r_{x},0,r_{z}\right)\in R^{3}:r_{x}^{2}+r_{z}^{2}=1\right\}\backslash \left\{\left(0,0,\pm 1\right)\right\}$$

and

(21) $$\varepsilon_{\rho }\left(S_{y};M\right)=0⟺\rho ≃r\in \left\{\left(0,r_{y},r_{z}\right)\in R^{3}:r_{y}^{2}+r_{z}^{2}=1\right\}\backslash \left\{\left(0,0,\pm 1\right)\right\},$$

the collections of which form the ‘great circles’ (orthodromes) of the Bloch sphere that passes through the eigenvectors of the observables to be measured and the ‘measurement observable’ $S_{z}$, barring the eigenvectors of the latter (i.e., the ‘north pole’ (0,0,1) and the ‘south pole’ (0,0,−1)) themselves (see Figure 3). Here, note that these two sets have empty intersection; this is to say that the errors of the measurement of the non-commutative observables $S_{x}$ and $S_{y}$ can never vanish simultaneously, which is indeed consistent with the (weaker form of the) uncertainty principle demonstrated in Section 4.2. In regard to this, it is illuminating to see that the error of the measurement of one of the target observables takes the maximal value whenever that of the other vanishes:

(22) $$\varepsilon_{\rho }\left(S_{x};M\right)=0⟹\varepsilon_{\rho }\left(S_{y};M\right)=\frac{\hbar }{2}$$

and

(23) $$\varepsilon_{\rho }\left(S_{x};M\right)=\frac{\hbar }{2}⟸\varepsilon_{\rho }\left(S_{y};M\right)=0.$$

Meanwhile, the errors $\varepsilon_{\rho }\left(S_{x};M\right)=\varepsilon_{\rho }\left(S_{y};M\right)=\hbar /2$ both take the maximal value over the ‘north pole’ and the ‘south pole’.

#### 6.2.2. Violation of the Naïve Lower Bound

The above observation reveals that the product of the errors $\varepsilon_{\rho }\left(S_{x};M\right)\;\varepsilon_{\rho }\left(S_{y};M\right)=0$ vanishes precisely over the region specified in (20) and (21). Combining this with the evaluation of the naïve lower bound $\left|{\langle \left[S_{x},S_{y}\right]\rangle }_{\rho }\right|/2=\left(\hbar /2\right)\;\left|r_{z}\right|$, one finds that its violation

(24) $$\left|{\langle \left[S_{x},S_{y}\right]\rangle }_{\rho }\right|/2>\varepsilon_{\rho }\left(S_{x};M\right)\;\varepsilon_{\rho }\left(S_{y};M\right)$$

can be attained over the said region for $r_{z}\neq 0$ (see also Section 4.2). In fact, without much difficulty, it is possible to analytically identify the exact domain over which the violation (24) occurs by means of Formulae (18) and (19). A detailed analysis of this is given elsewhere in another appropriate context.

#### 6.2.3. Evaluation of the Uncertainty Relation

The trade-off relation between the measurement errors (18) and (19) is quantitatively evaluated in the light of the new uncertainty relation (8). In this regard, the product of the errors is found to read

(25) $$\varepsilon_{\rho }\left(S_{x};M\right)\;\varepsilon_{\rho }\left(S_{y};M\right)={\left(\frac{\hbar }{2}\right)}^{2}\sqrt{\frac{r_{x}^{2}r_{y}^{2}}{{\left(1-r_{z}^{2}\right)}^{2}}+\left(1-\frac{r_{x}^{2}+r_{y}^{2}}{1-r_{z}^{2}}\right)}$$

by straightforward computation. As for the evaluation of its lower bound, the semiclassical contribution (9) is readily found to read

(26) $$R=-{\left(\frac{\hbar }{2}\right)}^{2}\frac{r_{x}r_{y}}{1-r_{z}^{2}}$$

by simple computation utilizing the pushforwards (16) and (17). The quantum contribution (10) of the lower bound can be obtained through the computation of the pullbacks of both the pushforwards (16) and (17) by the projection measurement associated with the ‘measurement observable’ $S_{z}$, thereby resulting in

(27) $$I={\left(\frac{\hbar }{2}\right)}^{2}\left(1-\frac{r_{x}^{2}+r_{y}^{2}}{1-r_{z}^{2}}\right)r_{z}.$$

Combining the results (26) and (27), one reveals the lower bound

(28) $$\sqrt{R^{2}+I^{2}}={\left(\frac{\hbar }{2}\right)}^{2}\sqrt{\frac{r_{x}^{2}r_{y}^{2}}{{\left(1-r_{z}^{2}\right)}^{2}}+{\left(1-\frac{r_{x}^{2}+r_{y}^{2}}{1-r_{z}^{2}}\right)}^{2}r_{z}^{2}}$$

of the product of the errors. The validity of the uncertainty relation (8) can be directly confirmed by Formulae (25) and (28), for indeed the former is never less than the latter for |r|≤1. In addition, they also allow for the characterization of the necessary and sufficient conditions for the equality to hold; the equality of the relation holds if and only if the quantum state over which the measurement is performed is pure (i.e., |r|=1), or in other words, the equality of the relation holds precisely over all the Bloch sphere (see Figure 3).

#### 6.2.4. The Semiclassical Contribution and the Reduction to the Simplified Form

**Figure 4:**
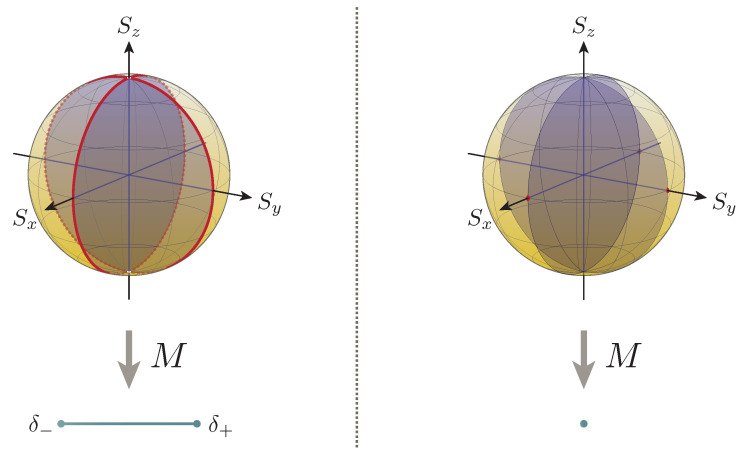
Reduction of the uncertainty relation to its simplified form. The region in which the semiclassical contribution R=0 to the lower bound vanishes, or equivalently, that in which the relation (8) reduces to its simplified form (11), is colored in violet. For this example, the conditions are the same for both the dichotomic measurement (**left**) and the trivial measurement (**right**). For the sake of comparison, the red regions over which the measurements are free from error are shown alongside (see Figure 3).

It is tempting to investigate the conditions under which the uncertainty relation (8) reduces to its simplified form (11). By construction, the reduction takes place precisely when the semiclassical contribution (9) to the lower bound vanishes, which happens under the conditions

(29) $$R=0⟺r_{x}=0\;\mathrm{or}\;r_{y}=0$$

for the current example, as one may directly find from Formula (26). The collection of all the quantum states over which the relation and its simplified form coincide can thus be depicted as the union of the two cross-sections of the ball that are respectively orthogonal to the *x* and *y* axes (see Figure 4).

Specifically, this reveals that the semiclassical contribution R≠0 is relevant over almost every state, thereby implying its general significance. In particular, in view of the fact that the quantum contribution (10) vanishes over all the Bloch sphere with the exception of the ‘north pole’ and ‘south pole’ (Formula (27) reveals

(30) $$I=0⟺\left|r\right|=1,r_{z}\neq \pm 1\;\mathrm{or}\;r_{z}=0$$

for the current example), the maximum tightness (i.e., attainment of the lower bound) of the relation over the sphere is actually almost solely due to the semiclassical contribution R, rendering it indispensable for the precise evaluation of the trade-off relation; in fact, the simplified form (11) merely yields the trivial evaluation $\varepsilon_{\rho }\left(S_{x};M\right)\;\varepsilon_{\rho }\left(S_{y};M\right)\geq 0$ over all the sphere but the ‘north pole’ and the ‘south pole’.

### 6.3. Trivial Measurements

Next, trivial measurements on two-state systems are investigated. As is demonstrated in Section 5, triviality of the measurement reduces the errors of the measurement of $S_{x}$ and $S_{y}$ to their respective standard deviations

(31) $$\varepsilon_{\rho }\left(S_{x};M\right)=\sigma_{\rho }\left(S_{x}\right)=\left(\frac{\hbar }{2}\right)\sqrt{1-r_{x}^{2}},\;\varepsilon_{\rho }\left(S_{y};M\right)=\sigma_{\rho }\left(S_{y}\right)=\left(\frac{\hbar }{2}\right)\sqrt{1-r_{y}^{2}},$$

which vanish if and only if the state over which the measurement is performed is one of their eigenstates (see Figure 3). Since the observables $S_{x}$ and $S_{y}$ have no common eigenstates, one finds that there exists no quantum state for which the errors (i.e., standard deviations) vanish simultaneously, which is of course just the well-known fact from elementary quantum mechanics; in the current context, this attests to the consistency with the uncertainty principle described in Section 4.2.

As for the quantitative evaluation of the trade-off relation between the measurement errors (i.e., standard deviations), recall that the relation (8) turns into the Schrödinger relation (12) for general mixed states when the measurement is trivial. The product of the errors thus reads

(32) $$\varepsilon_{\rho }\left(S_{x};M\right)\;\varepsilon_{\rho }\left(S_{y};M\right)=\sigma_{\rho }\left(S_{x}\right)\;\sigma_{\rho }\left(S_{y}\right)={\left(\frac{\hbar }{2}\right)}^{2}\sqrt{\left(1-r_{x}^{2}\right)\left(1-r_{y}^{2}\right)}$$

with the lower bound

(33) $$\sqrt{R^{2}+I^{2}}=\sqrt{{\left|{\langle \frac{\left\{S_{x},S_{y}\right\}}{2}\rangle }_{\;\rho }-{\langle S_{x}\rangle }_{\rho }{\langle S_{y}\rangle }_{\rho }\right|}^{2}+{\left|{\langle \frac{\left[S_{x},S_{y}\right]}{2i}\rangle }_{\;\rho }\right|}^{2}}={\left(\frac{\hbar }{2}\right)}^{2}\sqrt{r_{x}^{2}r_{y}^{2}+r_{z}^{2}}.$$

The validity of the relation for trivial measurements (i.e., the Schrödinger relation) can be directly confirmed by Formulae (32) and (33), for indeed the inequality

(34) $$\left(1-r_{x}^{2}\right)\left(1-r_{y}^{2}\right)=\left(1-{\left|r\right|}^{2}\right)+r_{x}^{2}r_{y}^{2}+r_{z}^{2}\geq r_{x}^{2}r_{y}^{2}+r_{z}^{2}$$

holds for |r|≤1. In addition, they also allow for the characterization of the necessary and sufficient conditions for the equality to hold; again, the equality of the relation holds if and only if the quantum state over which the measurement is performed is pure (i.e., |r|=1), or alternatively, the equality of the relation holds precisely over all the Bloch sphere (see Figure 3).

In passing, recall that, for projection measurements, the relation (8) reduces to that of trivial measurements (12) whenever the measurement is performed over the eigenstates of the corresponding ‘measurement observable’ (see Section 5). For the current example, this can be confirmed by the direct substitution of the parameters $\left(r_{x},r_{y},r_{z}\right)=\left(0,0,\pm 1\right)$ in (25) and (28) for the non-trivial projection measurement, as well as in (32) and (33) for trivial measurements; here, both cases are indeed found to yield the same (in)equality $\sigma_{\rho }\left(S_{x}\right)\;\sigma_{\rho }\left(S_{y}\right)={\left(\hbar /2\right)}^{2}=\sqrt{R^{2}+I^{2}}$.

### 6.4. Remarks on the Attainment of the Lower Bound

The uncertainty relation (8) has so far been demonstrated to attain the lower bound over all pure states regarding the measurement on two-state quantum systems, thereby offering virtually the tightest bound possible. However, it should be noted that the relation (8) is in general a non-trivial inequality, i.e., there are instances (of pure states) in which the equality does not hold, as should be the case. In order to avoid any such misconceptions, a simple counterexample that attests to this fact is included below.

One such physically relevant model would be the measurement on quantum harmonic oscillators. Since a proper treatment of unbounded observables defined on infinite-dimensional Hilbert spaces requires the introduction of additional mathematical tools and techniques that are beyond the scope of this paper, a more detailed analysis is reserved for another occasion. In what follows, the simplest of the settings, namely, the trivial measurements and the projection measurement associated with the Hamiltonian over the energy eigenstates, is investigated, in which cases the relation (8) reduces to the Schrödinger relation (12) (see Section 5). Below, one validates the basic fact that the Schrödinger relation does not necessarily attain the lower bound over the energy eigenstates.

For this, let $\hat{x}$ and $\hat{p}$ respectively denote the familiar position and momentum operators on the quantum system $H=L^{2}\left(R\right)$ of a one-dimensional non-relativistic free particle with mass *m*. The Hamiltonian

(35) $$H:=\frac{{\hat{p}}^{2}}{2m}+\frac{1}{2}m\omega^{2}{\hat{x}}^{2}$$

of the quantum harmonic oscillator with angular frequency ω (the symbol is not to be confused with that denoting an element of the sample space Ω) is well-known to be a self-adjoint operator whose spectrum consists solely of point spectrum (i.e., the set of eigenvalues). As is familiar, the eigenvalues $E_{n}=\hbar \omega \left(n+1/2\right)$, $n\in N_{0}$ are characterised by the non-negative integers, which are moreover all non-degenerated. The corresponding unit eigenfunctions are hereafter denoted by $\psi_{n}$, $n\in N_{0}$, disregarding the global phase.

One readily finds that the errors of both the measurement (i.e., standard deviations) of the observables $\hat{x}$ and $\hat{p}$ respectively read

(36) $$\varepsilon_{n}\left(\hat{x};M\right)=\sigma_{n}\left(\hat{x}\right)=\lambda \;\sqrt{n+\frac{1}{2}},\;\varepsilon_{n}\left(\hat{p};M\right)=\sigma_{n}\left(\hat{p}\right)=\frac{\hbar }{\lambda }\;\sqrt{n+\frac{1}{2}}$$

over the energy eigenstates, where the natural (characteristic) length $\lambda :=\sqrt{\hbar /(m\omega )}$ as well as the abbreviations $\varepsilon_{n}\left(A;M\right):=\varepsilon_{\psi_{n}}\left(A;M\right)$ and $\sigma_{n}\left(A\right):=\sigma_{\psi_{n}}\left(A\right)$ are adopted for better readability. Specifically, note that both the errors never vanish over the energy eigenstates (whereby the validity of the uncertainty principle described in Section 4.2 is also confirmed), which is of course just a rephrasing of the basic facts from elementary quantum mechanics.

Regarding the quantitative assessment of the relation, one immediately finds that the product of the errors reads

(37) $$\varepsilon_{n}\left(\hat{x};M\right)\;\varepsilon_{n}\left(\hat{p};M\right)=\sigma_{n}\left(\hat{x}\right)\;\sigma_{n}\left(\hat{p}\right)=\hbar \left(n+\frac{1}{2}\right).$$

As for the lower bound of the relation, one finds that the semiclassical contribution $R={\langle \left\{\hat{x},\hat{p}\right\}/2\rangle }_{n}-{\langle \hat{x}\rangle }_{n}{\langle \hat{p}\rangle }_{n}=0$ permanently vanishes over all the energy eigenstates, whereas the quantum contribution $I={\langle \left[\hat{x},\hat{p}\right]/2i\rangle }_{n}=\hbar /2$ gives the state-independent constant value, thereby resulting in

(38) $$\sqrt{R^{2}+I^{2}}=\sqrt{{\left|{\langle \frac{\left\{\hat{x},\hat{p}\right\}}{2}\rangle }_{\;n}-{\langle \hat{x}\rangle }_{n}{\langle \hat{p}\rangle }_{n}\right|}^{2}+{\left|{\langle \frac{\left[\hat{x},\hat{p}\right]}{2i}\rangle }_{\;n}\right|}^{2}}=\frac{\hbar }{2},$$

where the abbreviation ${\langle A\rangle }_{n}:={\langle A\rangle }_{\psi_{n}}$ is adopted for simplicity. From the above observations, the uncertainty relation for both measurements (i.e., the Schrödinger relation) is actually found to coincide with its simplified form (i.e., the Kennard–Robertson relation) over all the energy eigenstates, due to the absence of the semiclassical contribution R=0. In addition, the relation is found to attain the lower bound only over the ground state (n=0); otherwise, the relation is a strict inequality (n≥1).

## 7. Discussion

It is tempting to discuss some of the known uncertainty relations in the light of the new one. In this regard, the relation (8) has been already found to reduce to the Schrödinger relation (12)—thereby further leading to the Kennard–Robertson relation (1)—for the indeterminacy of quantum states as the notion of measurement degenerates into triviality (see Section 5).

As is presented in later publication, the framework [47] is found to entail Ozawa’s relation [9], as well as its recent modification [12]. More specifically, an enhancement of the relation (8) to accommodate joint measurability is available, which is found to be tighter than Ozawa’s relations. One shall find that whenever Ozawa’s error ε(A), which is accountable for POVM measurements with real outcomes Ω=R, is well-defined, so is the error $\varepsilon_{\rho }\left(A\right)$ introduced by the definition (6), further revealing that the former is never less than the latter. One then proves

(39) $$\varepsilon \left(A\right)\;\varepsilon \left(B\right)\geq \varepsilon_{\rho }\left(A\right)\;\varepsilon_{\rho }\left(B\right)\geq \sqrt{R^{2}+I^{2}}\geq \left|I\right|\geq \left|{\langle \left[A,B\right]\rangle }_{\rho }\right|/2-\varepsilon \left(A\right)\;\sigma_{\rho }\left(B\right)-\sigma_{\rho }\left(A\right)\;\varepsilon \left(B\right),$$

where R and I are the contributions to the lower bound of the product of the errors that respectively correspond to (9) and (10) regarding the enhancement of the relation (8). Here, note that the inequality consisting of the left- and right-most hand sides is equivalent to Ozawa’s, whereas the inequality in the middle is that corresponding to (the simplified form (11) of) the enhancement of the relation (8). An in-depth evaluation of Ozawa’s formulation in view of the new formalism [47], as well as the general proof of the evaluation (39), is beyond the scope of this paper, and will thus be given elsewhere. For now, some simple examples are given below that attest to the tightness for the special case in which both the observables are measured through a single common POVM measurement.

For this purpose, the previous model regarding the measurement on two-state quantum systems explored in Section 6 serves as a good example; note that this indeed falls into the category of POVM measurements with real outcomes. Under the same settings, one finds by simple computation that Ozawa’s errors regarding the measurement of each of the target observables $S_{x}$ and $S_{y}$ by the ‘measurement observable’ $S_{z}$ become the state-independent constant function $\varepsilon \left(S_{x}\right)=\varepsilon \left(S_{y}\right)=\sqrt{2}\;\left(\hbar /2\right)$, whereby the product of his errors reads $\varepsilon \left(S_{x}\right)\;\varepsilon \left(S_{y}\right)=2\;{\left(\hbar /2\right)}^{2}$. On the other hand, one immediately finds from Formula (25) that the product of the errors (6) is never greater than its half ${\left(\hbar /2\right)}^{2}\geq \varepsilon_{\rho }\left(S_{x}\right)\;\varepsilon_{\rho }\left(S_{y}\right)$, which is indeed consistent with the left-most instance

(40) $$\varepsilon \left(S_{x}\right)\;\varepsilon \left(S_{y}\right)>\varepsilon \left(S_{x}\right)\;\varepsilon \left(S_{y}\right)/2\geq \varepsilon_{\rho }\left(S_{x}\right)\;\varepsilon_{\rho }\left(S_{y}\right)$$

of the sequence of inequalities (39). Meanwhile, the existence of the common constant upper bound $\hbar /2\geq \sigma_{\rho }\left(S_{i}\right)$ of the standard deviations of each of the components of the spin-1/2 angular momentum leads to the evaluations $\varepsilon \left(S_{x}\right)\geq \sqrt{2}\;\sigma_{\rho }\left(S_{x}\right)$ and $\varepsilon \left(S_{y}\right)\geq \sqrt{2}\;\sigma_{\rho }\left(S_{y}\right)$, whereby the sum of the second and third terms of Ozawa’s inequality is found to be bounded from below by $\varepsilon \left(S_{x}\right)\;\sigma_{\rho }\left(S_{y}\right)+\sigma_{\rho }\left(S_{x}\right)\;\varepsilon \left(S_{y}\right)\geq 2\sqrt{2}\;\sigma_{\rho }\left(S_{x}\right)\;\sigma_{\rho }\left(S_{y}\right)\geq 2\sqrt{2}\;\left|{\langle \left[S_{x},S_{y}\right]\rangle }_{\rho }\right|/2$, in which the right-most inequality can be obtained by a straightforward application of the Kennard–Robertson inequality (1); this trivially validates the right-most instance

(41) $$\left|I\right|\geq 0\geq \left(1-2\sqrt{2}\right)\left|{\langle \left[S_{x},S_{y}\right]\rangle }_{\rho }\right|/2\geq \left|{\langle \left[S_{x},S_{y}\right]\rangle }_{\rho }\right|/2-\varepsilon \left(S_{x}\right)\;\sigma_{\rho }\left(S_{y}\right)-\sigma_{\rho }\left(S_{x}\right)\;\varepsilon \left(S_{y}\right)$$

of the sequence of inequalities (39).

In passing, one finds from the results obtained so far that the product of Ozawa’s errors reads twice the maximal value of the naïve lower bound

(42) $$\varepsilon \left(S_{x}\right)\;\varepsilon \left(S_{y}\right)=2\;{\left(\hbar /2\right)}^{2}>{\left(\hbar /2\right)}^{2}\geq \left|{\langle \left[S_{x},S_{y}\right]\rangle }_{\rho }\right|/2.$$

This further reveals, with additional simple computation, that the left- and right-hand sides of his inequality respectively have constant lower and upper bounds

(43) $$\varepsilon \left(S_{x}\right)\;\varepsilon \left(S_{y}\right)+\varepsilon \left(S_{x}\right)\;\sigma_{\rho }\left(S_{y}\right)+\sigma_{\rho }\left(S_{x}\right)\;\varepsilon \left(S_{y}\right)\geq \left(2+\sqrt{2}\right){\left(\hbar /2\right)}^{2}>{\left(\hbar /2\right)}^{2}\geq \left|{\langle \left[S_{x},S_{y}\right]\rangle }_{\rho }\right|/2,$$

thereby pointing to the existence of a gap of no less than $\left(1+\sqrt{2}\right){\left(\hbar /2\right)}^{2}$ lying between them. This specifically indicates that the product of Ozawa’s errors is incapable of the violation of the naïve lower bound $|{\langle \left[S_{x},S_{y}\right]\rangle }_{\rho }|/2$ for the current example, along with the fact that his relation never attains the lower bound; on the other hand, the relation (8) is capable of the violation (see Section 6.2.2) and attains the lower bound over all the Bloch sphere (see Section 6.2.3).

Trivial POVM measurements with real outcomes also serve as another elementary example that endorses the tightness of the relation (8). Provided that Ozawa’s error is well-defined for the given choice of trivial POVM measurement, one readily finds by definition that Ozawa’s error $\varepsilon \left(A\right)\geq \sigma_{\rho }\left(A\right)$ is never less than the standard deviation; this is indeed consistent with the aforementioned property that Ozawa’s error $\varepsilon \left(A\right)\geq \varepsilon_{\rho }\left(A\right)=\sigma_{\rho }\left(A\right)$ is never less than the error (6), combined with the fact that triviality of the measurement reduces the latter to the standard deviation (see Section 5). Thus for any such trivial POVM measurements, the Ozawa relation $\varepsilon \left(A\right)\;\varepsilon \left(B\right)+\varepsilon \left(A\right)\;\sigma_{\rho }\left(B\right)+\sigma_{\rho }\left(A\right)\;\varepsilon \left(B\right)\geq 3\;\sigma_{\rho }\left(A\right)\;\sigma_{\rho }\left(B\right)\geq 3\;\left|{\langle \left[A,B\right]\rangle }_{\rho }\right|/2\geq \left|{\langle \left[A,B\right]\rangle }_{\rho }\right|/2$ is found to be no tighter than three times the Kennard–Robertson relation, whereas the relation (8)—recall that the latter reduces to the Schrödinger relation (12) for general mixed states when the measurement is non-informative (see Section 5)—in fact becomes tighter than the Kennard–Robertson relation.
